# Supplementary material for: Dual-comb spectroscopic ellipsometry
Source: Nat Commun. 2017 Sep 20;8:610. doi: 10.1038/s41467-017-00709-y (PMC5606991; doi:10.1038/s41467-017-00709-y)
Supplement: Supplementary file 1 — Supplementary Information [file 41467_2017_709_MOESM1_ESM.pdf]

Description of Supplementary Files

File Name: Supplementary Information
Description: Supplementary Figures

File Name: Supplementary Movie 1
Description: Ellipsometric parameter Ψ of a Soleil-Babinet compensator observed with the dual-comb spectroscopic ellipsometry system (red dots) and by theoretical estimation (mesh surface) at a variety of relative wedge distances of the Soleil-Babinet compensator.

File Name: Supplementary Movie 2
Description: Ellipsometric parameter Δ of a Soleil-Babinet compensator observed with the dual-comb spectroscopic ellipsometry system (red dots) and by theoretical estimation (mesh surface) at a variety of relative wedge distances of the Soleil-Babinet compensator.

File Name: Supplementary Movie 3
Description: Ellipsometric parameter Ψ of a high-order wave plate obtained with the dual-comb spectroscopic ellipsometry system at a variety of rotation angles of the high-order wave plate.

File Name: Supplementary Movie 4
Description: Ellipsometric parameter Δ of a high-order wave plate obtained with the dual-comb spectroscopic ellipsometry system at a variety of rotation angles of the high-order wave plate.

File Name: Supplementary Movie 5
Description: Ellipsometric parameter Ψ of thin film samples obtained with the dual-comb spectroscopic ellipsometry system (red dots) and by theoretical estimation (mesh surface).

File Name: Supplementary Movie 6
Description: Ellipsometric parameter Δ of thin film samples obtained with the dual-comb spectroscopic ellipsometry system (red dots) and by theoretical estimation (mesh surface).

File Name: Peer Review File

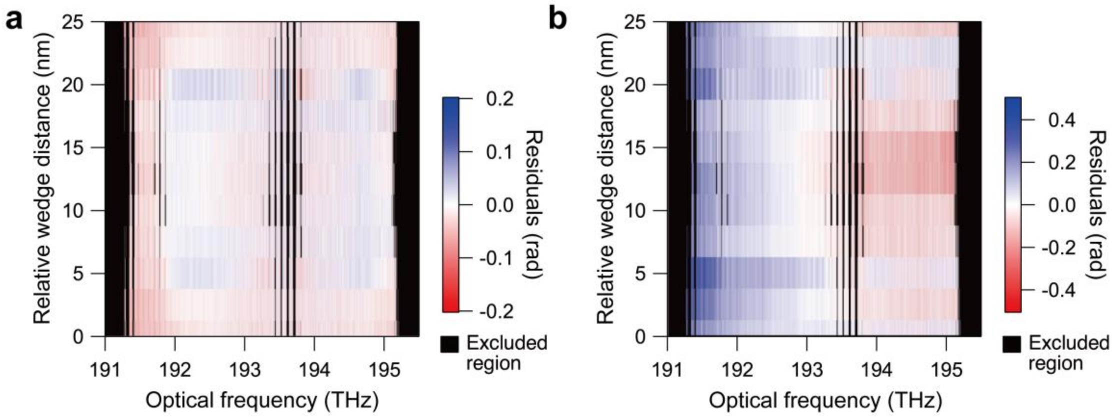

Supplementary Figure 1. Dual-comb spectroscopic ellipsometry estimation errors of the ellipsometric parameters for the Soleil-Babinet compensator. Dual-comb spectroscopic ellipsometry estimation error of (a) the amplitude ratio Ψ and (b) the phase difference Δ between the x- and y-polarization components of the polarization state of the incident light.

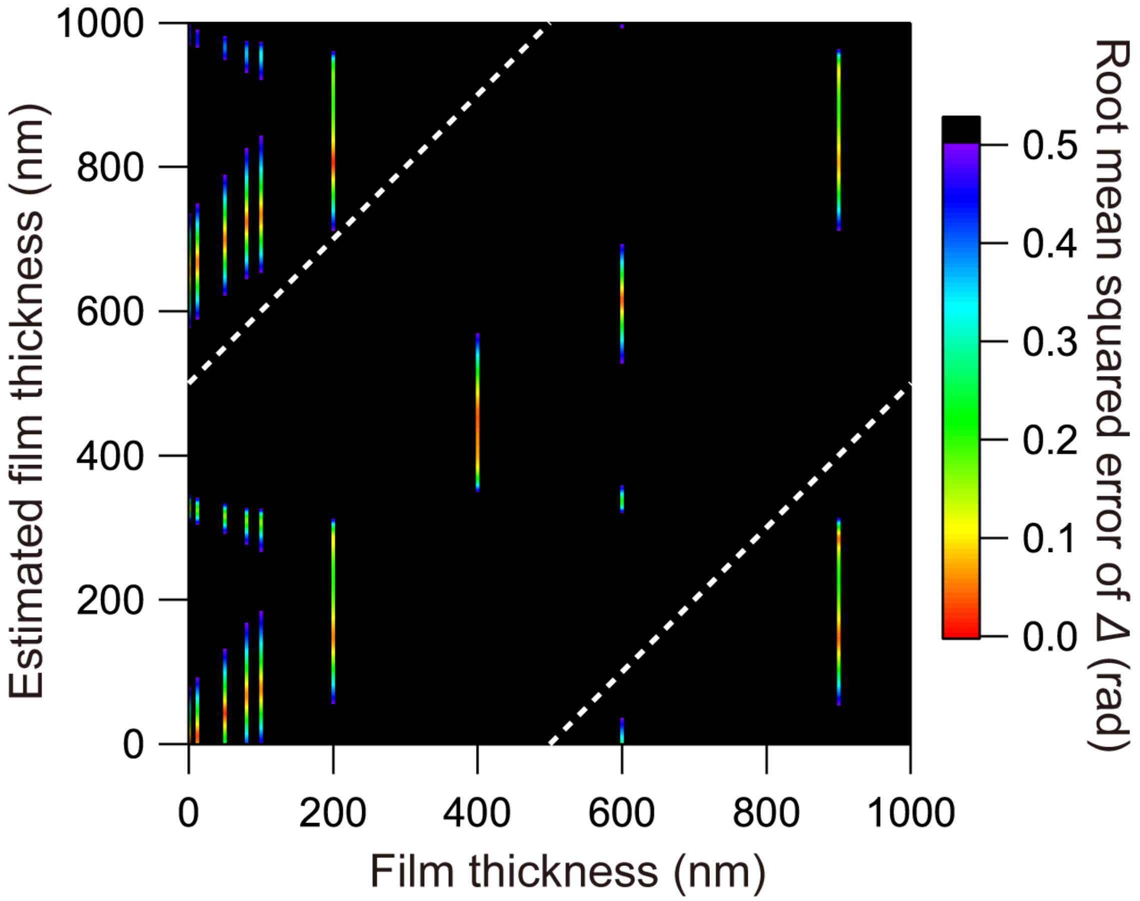

Supplementary Figure 2. Thickness measurement by the dual-comb spectroscopic ellipsometry system in terms of root mean squared error of the ellipsometric parameter *Δ*. Dashed lines indicate the predictable boundary at 500 nm from actual film thickness. The black region represents the root mean squared error of *Δ* that is equal to or larger than 0.5 rad.

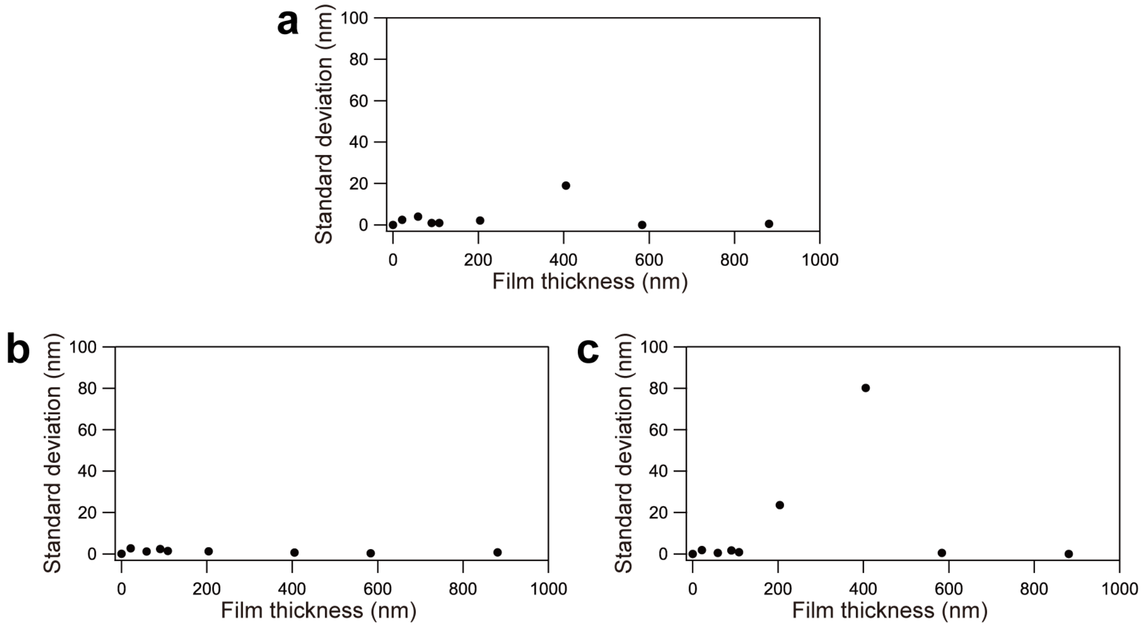

Supplementary Figure 3. Comparison of thickness determination precision of the dual-comb spectroscopic ellipsometry and conventional spectroscopic ellipsometry. (a) Thickness determination precision of the dual-comb spectroscopic ellipsometry with the wavelength range from 1514 to 1595 nm. (b) Thickness determination precision of conventional spectroscopic ellipsometry with the wavelength range from 1000 to 1689 nm. (c) Thickness determination precision of conventional spectroscopic ellipsometry with the wavelength range from 1514 to 1595 nm.
